# Metabolomics Approach Reveals the Effects of Breed and Feed on the Composition of Chicken Eggs

**DOI:** 10.3390/metabo9100224

**Published:** 2019-10-13

**Authors:** Tatsuhiko Goto, Hiroki Mori, Shunsuke Shiota, Shozo Tomonaga

**Affiliations:** 1 Research Center for Global Agromedicine, Obihiro University of Agriculture and Veterinary Medicine, Obihiro, Hokkaido 080-8555, Japan; 2 Department of Life and Food Sciences, Obihiro University of Agriculture and Veterinary Medicine, Obihiro, Hokkaido 080-8555, Japan; s27224@st.obihiro.ac.jp; 3 Graduate School of Agriculture, Kyoto University, Kyoto 606-8502, Japan; shiota.shunsuke.68v@st.kyoto-u.ac.jp (S.S.); shozo@kais.kyoto-u.ac.jp (S.T.)

**Keywords:** albumen, breed, chicken, feed, metabolome, yolk

## Abstract

Chicken eggs provide essential nutrients to consumers around the world. Although both genetic and environmental factors influence the quality of eggs, it is unclear how these factors affect the egg traits including egg metabolites. In this study, we investigated breed and feed effects on 10 egg traits, using two breeds (Rhode Island Red and Australorp) and two feed conditions (mixed feed and fermented feed). We also used gas chromatography–mass spectrometry (GC–MS/MS) to analyze 138 yolk and 132 albumen metabolites. Significant breed effects were found on yolk weight, eggshell weight, eggshell colors, and one albumen metabolite (ribitol). Three yolk metabolites (erythritol, threitol, and urea) and 12 albumen metabolites (erythritol, threitol, ribitol, linoleic acid, isoleucine, dihydrouracil, 4-hydroxyphenyllactic acid, alanine, glycine, N-butyrylglycine, pyruvic acid, and valine) were significantly altered by feed, and a significant interaction between breed and feed was discovered in one albumen metabolite (N-butyrylglycine). Yolk and albumin had higher levels of sugar alcohols when hens were fed a fermented diet, which indicates that sugar alcohol content can be transferred from diet into eggs. Linoleic acid was also enriched in albumen under fermented feed conditions. This study shows that yolk and albumen metabolites will be affected by breed and feed, which is the first step towards manipulating genetic and environmental factors to create “designer eggs.”

## 1. Introduction

Approximately 80 million tons of chicken eggs are produced every year, and they are a crucial source of animal protein in many developing countries [1,2]. Globally, 821 million people are malnourished [3], so international efforts to increase egg production are of high priority. In more developed countries, consumers are increasingly concerned with the quality of eggs and the bioavailability of favorable functional ingredients [4,5]. Increasing the quantity and quality of eggs around the world is key not only to alleviating hunger, but also to providing important dietary nutrients and keeping up with a rapidly changing international livestock industry. Currently, huge scientific effort is devoted to modifying agricultural produce traits to create “designer foods” [6]; eggs are no exception, and considerable effort is currently underway to modify egg traits and produce “designer eggs” to meet consumer demand [7,8].

Both genetic and environmental factors influence the quantity and quality of eggs [9,10,11]. Heritability estimates of egg traits such as overall weight, albumen weight, and yolk weight, are around 0.30–0.70 [12,13,14], which indicates that genetic factors are crucial to the regulation of egg traits. These general egg traits, which are mass and quality of egg components (yolk, albumen, and eggshell), are important for quality of egg itself as the product of egg market. Environmental factors including age, nutrition, stress, disease, medication, and production system also have important roles in modifying egg traits [9,10]. Thus, studies seeking to enhance egg traits should be cognizant of both genetic and environmental factors.

Egg enrichment with omega-3 polyunsaturated fatty acids (n-3 PUFA), which provide various human health benefits, is of current interest [15]. Evidence suggests that fatty acids quantity can be altered through changes to hens’ diet [16]. Other research suggests that responses to dietary enrichment and conversion into egg metabolites may be breed-specific [17]. Both breed and feed appear to regulate the abundance of metabolites in egg yolk and albumen. Recently, we also have reported that egg yolk amino acid was modified by both breed and feed [18]. This is very likely to be the tip of the iceberg regarding the effects of genetic and environmental factors on egg composition.

Metabolomics, the study of the set of metabolites present in various tissues, is used to identify novel metabolites or changes in metabolite ratios in tissues [19]. Metabolomic analyses are used in a variety of fields, including biomedicine [20,21], plant science [19], food science [22,23], and ecological/environmental science [24,25]. Typical metabolite analyses use gas chromatography–mass spectrometry (GC–MS), liquid chromatography–mass spectrometry (LC–MS), capillary electrophoresis–mass spectrometry (CE–MS), and nuclear magnetic resonance (NMR). Metabolome analyses have previously been used to identify changes caused by stress and/or feed in mice using CE–MS [26] and GC–MS [27], so we expect a metabolomics approach to provide useful insight into the role of breed and feed in determining egg composition. 

As studies into the metabolomes of livestock animals accumulate, a central database—the Livestock Metabolome Database—has been developed to compile this information. Research into cattle metabolic constitutes a large proportion of this work (76 of 149 articles), as does research into animal health, nutrition, and production (97 of 149 papers; [28]). For instance, effects of long-distance transportation in serum metabolites have been studied in cattle [29] and heat stress-induced metabolomic changes have been investigated in chicks [30]. Relatively few reports target non-major livestock breeds, and there is a need to populate the Livestock Metabolome Database with basic metabolome data for all livestock breeds.

In this study, we analyzed the yolk and albumen metabolome of eggs and general egg traits from two different breeds of hens under two different feed conditions. We used the GC–MS/MS technique to measure metabolome content, and tested how egg metabolites are influenced by specific genetic and environmental factors, i.e., breed and feed, respectively. 

## 2. Results

### 2.1. Egg Traits

To test the effects of breed and feed on egg traits, 10 traits were investigated (Table 1). Two-way mixed design analysis of variance (ANOVA) revealed a significant effect of breed on yolk weight (F_1,12_ = 8.098, *P* = 0.0147), eggshell weight (F_1,12_ = 7.287, *P* = 0.0193), lightness of eggshell color (F_1,12_ = 6.022, *P* = 0.0304), redness of eggshell color (F_1,12_ = 10.818, *P* = 0.0065), and yellowness of eggshell color (F_1,12_ = 14.394, *P* = 0.0026). Rhode Island Red (RIR) eggs showed higher yolk weight and lower eggshell weight than Australorp (AUS) eggs. Eggshell color in RIR eggs had less lightness, and more redness and yellowness than that of AUS. There were no significant effects of feed or interaction terms between breed and feed on any of the egg traits.

### 2.2. Egg Metabolite Traits

Egg metabolite traits from yolk (138 metabolites) and albumen (132 metabolites) were semi-quantified using GC−MS/MS. Full results were shown for yolk and albumen in Supplementary Tables S1 and S2, respectively. Controlling for multiple comparisons in each sample, some metabolites were found to be metabolome-wide significantly altered by breed and feed (*Q* < 0.1).

Albumen ribitol was significantly affected by breed (Table 2), with RIR eggs containing significantly higher ribitol levels than AUS eggs. Three metabolites in yolk and 12 metabolites in albumen had significant effects of feed (Table 3). Erythritol and threitol were significantly altered by feed in both the yolk and the albumen. Urea was altered in the yolk samples only, whereas isoleucine, dihydrouracil, linoleic acid, 4-hydroxyphenyllactic acid, alanine, glycine, N-butyrylglycine, pyruvic acid, ribitol, and valine were altered in albumen samples only. For threitol, erythritol, dihydrouracil, linoleic acid, pyruvic acid, and ribitol, the fermented feed group in both chicken breeds showed significantly higher metabolite content than mixed feed. On the other hand, the fermented feed group had significantly lower contents of urea, isoleucine, 4-hydroxyphenyllactic acid, alanine, glycine, N-butyrylglycine, and valine than the mixed feed group. There was a significant interaction between breed and feed on N-butyrylglycine in the albumen; N-butyrylglycine content in RIR chickens was higher with mixed feed than with fermented feed, but this effect was reversed in the AUS samples (Table 4).

## 3. Discussion

This study was designed to test the effects of breed and feed on 10 egg traits, 138 yolk metabolite traits, and 132 albumen metabolite traits, in RIR/AUS hens fed with either mixed feed or fermented feed. There were significant breed effects on yolk weight, eggshell weight, eggshell colors, and one albumen metabolite. Three yolk metabolites and 12 albumen metabolites were significantly altered by feed, and a significant interaction between breed and feed affected levels of one albumen metabolite. Using the metabolome technique, this study has demonstrated that certain egg properties, including metabolites in the yolk and albumen, can be changed by both genetic and environmental factors.

Since general egg traits, which are mass and quality of egg components (yolk, albumen, and eggshell), are crucial to maintain egg quality, we investigated 10 egg traits in this study. Egg traits including weight, length, width, albumen weight, and eggshell thickness showed no significant differences between RIR and AUS hens in this study, although AUS eggshell weight was higher than RIR. Average body weight at 35 weeks of age in RIR hens was more than twice that of AUS hens (3.69 and 1.58 kg in RIR and AUS, respectively). Yolk weight in RIR was significantly greater than that in AUS, corresponding to differences in body weight. We previously reported that Oh-Shamo (2.91 kg), and classical type of White Leghorn hens (1.54 kg) with average body weight at 36 weeks of age produced 53.8 ± 4.2 g and 47.4 ± 2.3 g of egg weight at 300 days of age, respectively [32,33]. Egg weight in AUS hens was 56.7 ± 4.9 g at around 300 days (41 weeks). Thus, AUS hens have a potential to produce eggs that are relatively larger than expected from their body weight [18]. On the other hand, significant breed effects were found on eggshell colors (lightness, redness, and yellowness), with RIR hens laying darker brown eggs than AUS hens. White to brown variation in eggshell color is a heritable quantitative trait [9,10,11], and eggshell colors of Oh-Shamo, White Leghorn, Hy-line Brown, and Onagadori chickens were reported [33,34,35]. Comparing this study to previous work, eggs appear to decrease in color from brown to white in the order of Hy-Line Brown > RIR > Oh-Shamo > AUS > Onagadori > White Leghorn [33,34,35]. Further efforts must be needed to reveal the genetic basis of eggshell coloration in chickens using population genomics and genome-wide scan. 

The present metabolome analyses revealed significant changes in the amount of sugar alcohols (polyols) present in eggs. Erythritol and threitol in the yolk and albumen appear to be affected by dietary changes, specifically moving to a fermented diet. Sugar alcohols are naturally found in small quantities in fruits, vegetables, mushrooms, and fermented foods such as wine, beer, sake, and soy sauce [36], and can be produced by several yeasts and fungi [37]. The fermented feed used in this study was made with wheat, pumpkin, yam, soybean, potato, rice bran, fish meal, beet lees, scallop shell, and other materials, and lactic acid bacteria were used to ferment the mixture [18]; the amount of sugar alcohols in fermented feed would be higher than in mixed feed. Eggs produced under the fermented feed treatment had high sugar alcohol content, which is likely due to the transfer of these nutrients from feed to eggs. The same trend was also apparent for ribitol, which was found at a higher concentration in eggs produced on the fermented diet. There was also a significant breed effect, and ribitol levels in RIR eggs were higher than those of AUS eggs. These results indicate that different genetic backgrounds can affect the transfer of nutrients into eggs. 

After 7 weeks on the fermented feed treatment, erythritol contents in both the yolk and albumen were significantly higher than on mixed feed. Erythritol is a four-carbon sugar alcohol (polyol) that has a sweetness 60% to 80% that of sucrose, and is used as a low-calorie sweetener [38]. This may indicate that yolk and albumen taste are affected by feed type. In humans, erythritol is fast absorbed through the small intestine, and >90% is excreted intact in urine. The remaining 10% is fermented in the large intestine by colonic microorganisms [36]. Polyols act as probiotics like a fiber, and can help normalize intestine function [36]. There are massive evidences that the intake of erythritol would not cause adverse effects in humans [38]. Artificial sweeteners and/or polyols may be helpful in diabetes and weight control, and eggs enriched with erythritol may be useful as a functional food. 

Linoleic acid levels in egg albumen were significantly increased by the fermented feed diet. Linoleic acid is a long-chain polyunsaturated fatty acid (PUFA), and an essential nutrient in wide range of animals including humans. PUFA is vital for body functions and plays an important role in the formation and functioning of cell membranes, cell physiology, signaling, immunity, and reproduction [39]. Many animals lack the ability to synthesize linoleic acid de novo, so dietary intake of linoleic acid is necessary [39]. Enrichment of eggs with PUFAs, including linoleic acid, is relatively common and increases the nutritional functionality of enriched eggs [15]. Linoleic acid is found at relatively high levels in seed oils [40]. Fermented feed may derive its high linoleic acid content from its constituent plant matter, which includes pumpkin seeds, or from its relatively high proportion of crude fat. However, although isoleucine, alanine, glycine, and valine levels in the fermented feed were also higher than those of mixed feed, albumin isoleucine, alanine, glycine, and valine contents of eggs under the fermented feed diet were significantly lower than those of the mixed feed diet. More works are required to understand the changes to these amino acids levels in albumen. 

There was a significant interaction between breed and feed on albumin N-butyrylglycine levels. N-butyrylglycine is an acylglycine [41], which is formed by the conjugation of acyl-CoA esters with glycine [42]. Acylglycines are used as diagnostic markers of inborn errors of metabolism [41], and previous studies have shown that acylglycine levels are sensitive in the urine of spontaneously hypertensive rats on a high-fat diet [42]. In this study, we found that acylglycine content was higher under the mixed feed treatment than fermented feed in RIR hens, but the opposite was true for AUS hens. Since N-butyrylglycine is involved in fatty acid metabolism [42], this may indicate that the combination of breed and feed can affect fatty acid metabolism. Actually, linoleic acid in albumen was significantly altered in this study. Such interaction effects of breed and feed have been documented in the enriched fatty acid levels of yolks in other studies [17]. In the modern chicken industry, hybrid chickens rather than pure breeds are often used for egg and meat production. This study indicates that the combination of breed and feed should be considered to modulate metabolite levels in yolk and albumen.

In conclusion, we found significant breed and feed effects on yolk weight, eggshell weight, eggshell colors, and several metabolites in the yolk and albumen. Feed alone had a notable impact on sugar alcohol and fatty acid levels, which were enriched in both yolk and albumen under the fermented feed treatment. We illustrated that both genetic and environmental factors are critical to determining egg composition and should be considered in the efforts to meet consumer needs and develop nutritionally functional designer eggs.

## 4. Materials and Methods 

### 4.1. Study Animals

Rhode Island Red (RIR; *n* = 5) and Australorp (AUS; *n* = 5) hens were procured from the Animal Research Center of the Hokkaido Research Organization, Japan. They were introduced to the experimental farm of the Obihiro University of Agriculture and Veterinary Medicine, Japan, at 22 weeks of age. After introduction, all hens were reared in individual cages under a photoperiod cycle of 16 h light and 8 h dark, with free access to diet and water. Body weight at 35 weeks of age was 3.69 ± 0.57 and 1.58 ± 0.09 kg (mean ± standard deviation) for RIR and AUS, respectively (F_1,8_ = 67.324, *P* = 3.6 × 10^−5^). Animal management was carried out following the Guide for the Use of Experimental Animals in Universities (The Ministry of Education, Science, Sports, and Culture, Tokyo, Japan 1987) and the Standards Related to the Care and Management of Experimental Animals (Prime Ministers’ Office, Tokyo, Japan, 1980). This study (authorization number 18–15) was approved from the Experimental Animal Committee of the Obihiro University of Agriculture and Veterinary Medicine.

### 4.2. Experimental Conditions and Sampling

**Figure 1:**
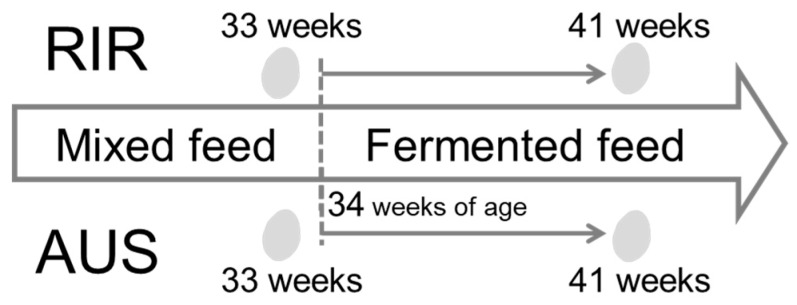
Experimental design. Eggs from Rhode Island Red (RIR) and Australorp (AUS) hens, which had been fed mixed feed, were collected at 33 weeks of age (mixed feed). Feed was switched at 34 weeks of age to fermented feed. Eggs were again collected at 41 weeks of age (fermented feed). Five eggs were collected at each stage from each breed; egg traits and yolk and albumen metabolite traits were measured in the 20 eggs. These data were analyzed using two-way mixed design analysis of variance (ANOVA) with breed group as the between-subjects factor and feed group as the within-subject factor.

To test the effects of breed and feed on egg metabolome, RIR and AUS hens were reared under two different feed conditions. Mixed feed (Rankeeper; Marubeni Nisshin Feed Co., Ltd., Tokyo, Japan) was provided for 11 weeks, from introduction until 33 weeks of age. Fermented feed (Kusanagi Farm Limited Company, Obihiro, Japan) was provided for 9 weeks, from 34 weeks of age until the end of the experiment. The fermented feed was made from many feed materials such as potato peel, cottons and seeds of pumpkin, sake lees, and wheat, especially using a silage preparation additive, WS360 (Protocol Japan Ltd., Obihiro, Japan), which contains lactic acid bacteria and cellulolytic enzyme [18]. The ingredients of both mixed and fermented feeds were analyzed at the Institute of Chemurgy, in the Tokachi Federation of Agricultural Cooperatives, Japan (Supplementary Table S3). Eggs from each breed (RIR and AUS) were collected at two stages: at the end of the mixed feed period (33 weeks of age) and near the end of the fermented feed period (41 weeks of age, see Figure 1). Five eggs were collected from each breed at each stage, totaling 20 eggs over the course of the study. 

### 4.3. Measuring Egg Properties 

Ten egg traits were measured, including egg weight, length of the long axis, length of the short axis, eggshell weight, yolk weight, albumen weight, eggshell thickness, and eggshell lightness (L*), redness (a*), and yellowness (b*). Size was measured using a digital caliper (P01 110–120; ASONE, Japan). Eggshell colors were measured using a chromameter (CR-10 Plus Color Reader; Konica Minolta Japan, Inc., Tokyo, Japan), and eggshell thickness was measured with a Peacock dial pipe gauge P-1 (Ozaki MFG Co., Ltd., Tokyo, Japan). After weighing, yolk and albumen were kept separately at −30 °C pending further analysis.

### 4.4. Metabolomic Analysis of Egg Yolk and Albumen 

Prior to metabolomic analysis, yolk and albumen samples were dried using a freeze dryer (FD-550P, Eyela; Tokyo Rikakikai Co. Ltd., Tokyo, Japan). Samples were crushed into a powder form, and 10 mg of each sample was used for metabolomic analysis. Powder samples were mixed with 250 μL of pretreatment liquid (methanol/H_2_O/chloroform 5:2:2) and 5 μL of internal standard solution (2-isopropylmalic acid, 1 mg/mL). After vortex mixing, the sample tubes were shaken at 1200 rpm at 37 °C for 30 min under dark light conditions. After centrifugation at 16,000× *g*, 4 °C for 5 min, the supernatants (160 μL) were collected and placed in new tubes with 200 μL of H_2_O and mixed using a vortex. The tubes were again centrifuged at 16,000× *g*, 4 °C for 5 min. The supernatants (200 μL) were collected into new tubes and centrifuged at room temperature in a vacuum (Centrifugal Evaporator RD400; Yamato Scientific Co. Ltd., Tokyo, Japan) for 20 min. Then, the tubes were placed in −80 °C until freezing. The tubes were centrifuged at room temperature in a vacuum for 7−8 h. Methoxyamine hydrochloride in pyridine (20 mg/mL, 40 μL) was added to each tube and mixed using the vortex. The tubes were shaken for 90 min at 1200 rpm, 30 °C, under dark conditions, for oximation. N-methyl-N-trimethylsilyltrifluoroacetamine (MSTFA; 20 μL) was added to each tube and vortex-mixed. The tubes were shaken at 1200 rpm at 37 °C for 45 min in the dark to prepare trimethylsilyl (TMS) derivatives. GC−MS/MS analyses were carried out using a GCMS-TQ8050 (Shimadzu, Kyoto, Japan) with BPX-5 column (Length 30 m; 0.25 mm I.D.; df = 0.25 μm) (SGE, Melbourne, Australia) according to the method in Smart Metabolites Database (Shimadzu, Kyoto, Japan).

Data processing was performed with Smart Metabolites Database (Shimadzu, Kyoto, Japan), MS-DIAL ver 3.08 [43] and MRMPROBS program ver. 2.42 [44]. Peaks of 40 samples (20 eggs × 2 samples) were recorded over the mass range 45−600 *m*/*z*. Peaks were automatically detected via MS-DIAL with peak detection options that minimum peak height is 2000. A data quality check was conducted using thresholds, which are −10 < RI < 10, dot prod > 0.8, and presence > 0.6 and then manually checked. Finally, 138 and 132 metabolites for yolk and albumen were identified, respectively. Relative quantity of metabolites was calculated using the peak area of each metabolite relative to an internal standard (2-isopropylmalic acid). 

### 4.5. Statistical Methods

To identify the effects of breed and feed on egg properties, data were analyzed using a two-way mixed-design analysis of variance (ANOVA) with breed group (RIR and AUS) as the between-subjects factor and feed group (mixed feed and fermented feed) as the within-subject (repeated) factor [18,45,46,47]. Main- and interaction-effects of breed and feed on egg properties were tested (*P* < 0.05). Data are presented as the mean and standard deviation. Statistical analyses were conducted using R [48]. In metabolites in particular, individual values were standardized with their mean and standard deviation. 

To control the *P* value for multiple comparisons in metabolites, the false discovery rate was determined using the approach of Benjamini and Hochberg [49]. The *P* values were adjusted using the Benjamini−Hochberg correction, and post-adjustment are referred to as *Q* values. The metabolome-wide significance threshold was set at *Q* < 0.1, following previous studies [26,27,50,51].

## Figures and Tables

**Table 1:** Egg traits at two stages in Rhode Island Red and Australorp.

Traits	Rhode Island Red (RIR)		Australorp (AUS)		*P*-Value from ANOVA Mixed Design		
	Mixed	Fermented	Mixed	Fermented	Main-Effect		Interaction-Effect
	33 Weeks	41 Weeks	33 Weeks	41 Weeks	Breed	Feed	Breed × Feed
Egg weight (g)	54.2 ± 2.0	55.5 ± 2.5	52.6 ± 4.7	56.7 ± 4.9	0.068	0.573	0.918
Length of long axis of the egg (mm)	56.2 ± 2.4	57.8 ± 1.0	54.8 ± 2.1	57.8 ± 1.6	0.067	0.421	0.563
Length of short axis of the egg (mm)	42.0 ± 0.9	41.8 ± 1.0	41.4 ± 1.4	41.2 ± 1.2	0.065	0.974	0.953
Yolk weight (g)	15.5 ± 0.5	17.0 ± 1.2	13.4 ± 1.1	15.5 ± 1.1	0.015	0.287	0.826
Eggshell weight (g)	6.0 ± 0.4	6.7 ± 0.5	6.8 ± 0.8	6.3 ± 1.4	0.019	0.657	0.186
Albumen weight (g)	29.7 ± 1.7	29.5 ± 2.0	29.5 ± 3.0	31.1 ± 2.7	0.360	0.404	0.971
Eggshell thickness (mm)	0.38 ± 0.01	0.40 ± 0.05	0.42 ± 0.03	0.38 ± 0.03	0.227	0.725	0.667
Eggshell color L *	62.5 ± 5.0	66.3 ± 5.4	68.5 ± 2.0	75.5 ± 1.5	0.030	0.705	0.950
Eggshell color a *	14.3 ± 2.9	12.2 ± 2.6	9.5 ± 1.3	6.3 ± 0.7	0.006	0.376	0.600
Eggshell color b *	22.3 ± 3.5	20.6 ± 3.0	15.2 ± 1.8	12.5 ± 2.1	0.003	0.453	0.886

**Table 2:** Metabolite with breed-induced changes (*Q* < 0.1).

Metabolite	HMDB ^1^	Relative Area (Mean ± Standard Deviation (SD))												Mixed-Design ANOVA		
		RIR			RIR			AUS			AUS			Breed		
		Mixed			Fermented			Mixed			Fermented			*P*-Value	*Q*-Value	
Albumen_Ribitol	HMDB0000508	−0.17	±	0.65	0.59	±	0.49	−0.84	±	1.27	0.42	±	0.59	0.0005	0.0628	*

^1^ The Human Metabolome Database [31]. * *Q* < 0.1.

**Table 3:** Metabolites with feed-induced changes (*Q* < 0.1).

Metabolite	HMDB ^1^	Relative Area (Mean ± SD)												Mixed Design ANOVA		
		RIR			RIR			AUS			AUS			Feed		
		Mixed			Fermented			Mixed			Fermented			*P*-Value	*Q*-Value	
Yolk_Urea	HMDB0000294	0.47	±	0.22	−0.78	±	0.56	0.81	±	1.24	−0.50	±	0.42	0.002	0.085	*
Yolk_Threitol	HMDB0004136	−0.84	±	0.27	0.89	±	0.33	−1.02	±	0.13	0.97	±	0.37	0.001	0.082	*
Yolk_Erythritol	HMDB0002994	−0.77	±	0.34	0.93	±	0.45	−1.05	±	0.09	0.89	±	0.39	0.001	0.075	*
Albumen_Isoleucine	HMDB0000172	0.26	±	0.77	−0.89	±	0.63	1.20	±	0.41	−0.57	±	0.39	0.001	0.020	*
Albumen_Dihydrouracil	HMDB0000076	−0.57	±	0.93	0.69	±	0.93	−0.77	±	0.48	0.65	±	0.43	0.000	0.001	*
Albumen_Erythritol	HMDB0002994	−0.67	±	0.20	1.13	±	0.72	−1.04	±	0.12	0.58	±	0.30	0.001	0.017	*
Albumen_Linoleic acid	HMDB0000673	−0.02	±	0.88	0.81	±	1.34	−0.75	±	0.32	−0.04	±	0.25	0.000	0.008	*
Albumen_4-Hydroxyphenyllactic acid	HMDB0000755	0.41	±	0.79	−1.05	±	0.52	1.05	±	0.43	−0.40	±	0.57	0.007	0.091	*
Albumen_Alanine	HMDB0000161	0.54	±	1.29	−0.75	±	0.39	0.85	±	0.55	−0.64	±	0.18	0.007	0.082	*
Albumen_Glycine	HMDB0000123	0.44	±	1.28	−0.70	±	0.73	0.83	±	0.42	−0.57	±	0.25	0.004	0.066	*
Albumen_N-Butyrylglycine	HMDB0000808	0.12	±	0.78	−0.34	±	0.72	−0.04	±	1.02	0.26	±	1.24	0.000	0.007	*
Albumen_Pyruvic acid	HMDB0000243	−0.06	±	1.36	0.19	±	0.99	−0.17	±	1.07	0.04	±	0.39	0.005	0.083	*
Albumen_Ribitol	HMDB0000508	−0.17	±	0.65	0.59	±	0.49	−0.84	±	1.27	0.42	±	0.59	0.007	0.083	*
Albumen_Threitol	HMDB0004136	−0.70	±	0.17	1.11	±	0.74	−1.01	±	0.14	0.59	±	0.34	0.001	0.020	*
Albumen_Valine	HMDB0000883	0.54	±	1.27	−0.77	±	0.36	0.89	±	0.49	−0.67	±	0.10	0.006	0.088	*

^1^ The Human Metabolome Database [31]. * *Q* < 0.1.

**Table 4:** Metabolite with breed × feed-induced changes (*Q* < 0.1).

Metabolite	HMDB ^1^	Relative Area (Mean ± SD)												Mixed Design ANOVA		
		RIR			RIR			AUS			AUS			Breed × Feed		
		Mixed			Fermented			Mixed			Fermented			*P*-Value	Q-Value	
Albumen_N-Butyrylglycine	HMDB0000808	0.12	±	0.78	−0.34	±	0.72	−0.04	±	1.02	0.26	±	1.24	0.0000	0.0048	*

^1^ The Human Metabolome Database [31]. * *Q* < 0.1.

## Supplementary Materials

The following are available online at https://www.mdpi.com/2218-1989/9/10/224/s1: Table S1: All yolk metabolites, Table S2: All albumen metabolites, Table S3: Ingredient analysis of mixed feed and fermented feed.
