# Supplementary material for: Metabolomics Approach Reveals the Effects of Breed and Feed on the Composition of Chicken Eggs
Source: Metabolites. 2019 Oct 13;9(10):224. doi: 10.3390/metabo9100224 (PMC6835386; doi:10.3390/metabo9100224)
Supplement: Supplementary file 1 [file metabolites-09-00224-s001.pdf]

Type of the Paper (Article)

Metabolomics approach reveals the effects of breed and feed on the composition of chicken eggs

Tatsuhiko Goto [1,2,*], Hiroki Mori [2], Shunsuke Shiota [3], Shozo Tomonaga [3]

[1] Research Center for Global Agromedicine, Obihiro University of Agriculture and Veterinary Medicine, Obihiro, Hokkaido 080-8555, Japan
[2] Department of Life and Food Sciences, Obihiro University of Agriculture and Veterinary Medicine, Obihiro, Hokkaido 080-8555, Japan
[3] Graduate School of Agriculture, Kyoto University, Kyoto 606-8502, Japan
* Correspondence: tats.goto@obihiro.ac.jp; Tel.: +81-155-49-5426

Received: date; Accepted: date; Published: date

Supplementary Table 1. All yolk metabolites.

Yolk metabolite	Relative area (mean ± SD)				Two-way mixed design ANOVA					
	RIR	RIR	AUS	AUS	Breed		Feed		Breed*Feed	
	Mixed	Fermented	Mixed	Fermented	p-value	q-value	p-value	q-value	p-value	q-value
1-Hexadecanol-TMS	-0.26 ± 0.53	-0.22 ± 0.40	0.45 ± 0.77	0.03 ± 1.57	0.0620	0.4503	0.1008	0.9274	0.0362	1.6652
2-Aminoadipic-acid-3TMS	-0.40 ± 1.01	-0.47 ± 0.90	0.22 ± 0.42	0.65 ± 0.97	0.0753	0.4723	0.8859	1.0449	0.3866	1.5243
2-Aminobutyric-acid-2TMS	-0.99 ± 0.17	0.78 ± 0.88	-0.51 ± 0.29	0.73 ± 0.72	0.1200	0.5710	0.7650	1.1113	0.8390	0.9896
2-Aminoethanol-3TMS	-0.64 ± 0.84	-0.06 ± 1.30	0.23 ± 0.74	0.47 ± 0.39	0.3580	0.7968	0.9070	0.9934	0.7330	1.0761
2-Aminoisobutyric-acid-2TMS	0.05 ± 1.45	-0.16 ± 0.55	0.19 ± 1.05	-0.09 ± 0.46	0.2860	0.7894	0.0181	0.4996	0.2860	1.8794
2-Aminopimelic-acid-3TMS	0.20 ± 0.59	0.69 ± 0.89	-0.53 ± 0.84	-0.37 ± 1.01	0.8020	0.9301	0.8430	1.0673	0.3080	1.7710
2-Deoxyuridine-3TMS	0.06 ± 1.27	-0.38 ± 0.84	0.48 ± 0.67	-0.17 ± 0.80	0.5210	0.8264	0.1780	0.9098	0.3890	1.4509
2-Hydroxyglutaric-acid-3TMS	-0.61 ± 1.11	0.95 ± 0.48	-0.14 ± 0.70	-0.20 ± 0.71	0.4314	0.8156	0.0072	0.2467	0.9639	0.9853
2-Ketoadipic-acid-meto-2TMS(2)	-0.77 ± 1.02	-0.38 ± 0.52	0.47 ± 0.84	0.67 ± 0.61	0.3850	0.7930	0.6940	1.1267	0.6460	1.1578
2-Ketoisocaproic-acid-meto-TMS(2)	-0.43 ± 1.45	-0.01 ± 0.74	0.02 ± 0.70	0.42 ± 0.54	0.4810	0.8621	0.1380	1.0023	0.8690	0.9911
2-Propyl-5-hydroxy-pentanoic-acid-2TMS	0.24 ± 1.63	-0.06 ± 0.66	0.17 ± 0.66	-0.35 ± 0.22	0.7120	0.8932	0.1280	1.0391	0.7810	1.0265
3-Aminoglutaric-acid-3TMS	-1.02 ± 0.66	0.12 ± 0.64	0.34 ± 1.06	0.56 ± 0.60	0.2750	0.8074	0.7030	1.1151	0.6290	1.1730
3-Aminoisobutyric-acid-2TMS	-0.80 ± 0.60	-0.18 ± 0.61	0.38 ± 1.26	0.60 ± 0.54	0.9770	1.0062	0.7830	1.1026	0.9220	1.0179
3-Aminopropanoic-acid-3TMS	-0.97 ± 0.60	1.01 ± 0.55	-0.51 ± 0.66	0.47 ± 0.52	0.3110	0.8253	0.0430	0.5934	0.5070	1.3719
3-Hydroxybutyric-acid-2TMS	-0.26 ± 0.67	0.78 ± 1.37	-0.26 ± 0.66	-0.26 ± 0.47	0.9960	1.0033	0.7480	1.1099	0.6100	1.2379
3-Hydroxyglutaric-acid-3TMS	0.16 ± 0.82	0.03 ± 1.14	-0.40 ± 0.82	0.20 ± 0.96	0.7330	0.8952	0.2570	1.0431	0.7900	1.0002
3-Hydroxyisobutyric-acid-2TMS	0.15 ± 1.15	0.50 ± 1.19	-0.41 ± 0.55	-0.25 ± 0.51	0.7540	0.8970	0.6050	1.1437	0.5770	1.3053
3-Methyl-2-oxovaleric-acid-meto-TMS(1)	0.15 ± 1.40	0.08 ± 0.72	0.03 ± 0.76	-0.27 ± 0.80	0.0130	0.8970	0.0370	0.5673	0.1550	1.7825
3-Sulfinoalanine-3TMS	0.25 ± 0.66	0.30 ± 1.52	-0.17 ± 0.48	-0.38 ± 0.70	0.0546	0.4432	0.0256	0.4416	0.1732	1.8386
4-Aminobutyric-acid-3TMS	-0.56 ± 0.86	-0.36 ± 0.62	0.70 ± 0.64	0.21 ± 1.13	0.1290	0.5934	0.3170	1.0416	0.3880	1.4873
4-Hydroxyphenyllactic-acid-3TMS	-0.78 ± 0.90	-0.08 ± 0.92	0.12 ± 0.80	0.74 ± 0.58	0.3700	0.8105	0.6990	1.1217	0.8870	0.9952
4-Hydroxyproline-3TMS	-0.73 ± 0.27	1.18 ± 0.95	-0.64 ± 0.29	0.19 ± 0.60	0.0632	0.4361	0.2343	1.0430	0.3544	1.8114
5-Aminovaleric-acid-3TMS	0.22 ± 0.33	-0.36 ± 0.68	1.17 ± 0.33	-1.04 ± 0.70	0.4920	0.8280	0.5600	1.1709	0.7100	1.0535
5-Oxoproline-2TMS	-0.77 ± 0.68	0.12 ± 0.96	0.04 ± 0.88	0.61 ± 0.81	0.4710	0.8552	0.9420	1.0000	0.6150	1.1954
Adenine-2TMS	-0.38 ± 1.01	-0.30 ± 0.76	0.17 ± 0.97	0.51 ± 0.87	0.0146	0.6716	0.1672	1.0032	0.8389	0.9980
Alanine-2TMS	-0.95 ± 0.36	-0.05 ± 0.66	0.28 ± 0.96	0.71 ± 0.91	0.0478	0.4712	0.8753	1.0504	0.9932	0.9932
Allose-meto-5TMS(1)	-0.49 ± 1.48	-0.13 ± 0.76	0.33 ± 0.73	0.28 ± 0.28	0.6060	0.8711	0.7860	1.0956	0.7590	1.0269
Arabitol-5TMS	0.58 ± 1.83	-0.22 ± 0.03	-0.26 ± 0.05	-0.10 ± 0.04	0.5450	0.8451	0.1500	1.0350	0.6460	1.1730
Arginine-3TMS	-0.71 ± 0.69	-0.36 ± 0.41	0.92 ± 1.24	0.15 ± 0.35	0.0155	0.5348	0.9846	0.9846	0.1788	1.7625
Asparagine-3TMS	-0.92 ± 0.93	-0.26 ± 0.54	0.47 ± 0.87	0.70 ± 0.51	0.3530	0.8257	0.9550	0.9984	0.7610	1.0196
Aspartic-acid-3TMS	-0.90 ± 0.48	0.41 ± 0.94	0.18 ± 1.00	0.32 ± 0.76	0.1510	0.6129	0.5650	1.1466	0.5280	1.3748
Benzoic-acid-TMS	-0.50 ± 0.61	0.72 ± 0.74	0.27 ± 1.07	-0.49 ± 0.80	0.7339	0.8884	0.3614	1.1082	0.0033	0.4485

Supplementary Table 1. (Continued)

Cadaverine-4TMS	-0.66	±	0.51	-0.36	±	0.45	-0.08	±	0.91	1.10	±	0.85	0.2680	0.8219	0.6160	1.1334	0.7550	1.0316
Caproic-acid-TMS	-0.69	±	0.52	0.93	±	1.20	0.17	±	0.25	-0.42	±	0.69	0.7440	0.8928	0.5430	1.2489	0.1360	2.3460
Cholesterol-TMS	0.72	±	1.74	-0.30	±	0.04	-0.32	±	0.01	-0.09	±	0.25	0.5750	0.8625	0.1500	0.9857	0.5010	1.4110
Citric-acid-4TMS	0.13	±	0.65	0.28	±	1.00	-0.19	±	1.16	-0.23	±	0.92	0.9310	0.9960	0.9790	0.9934	0.2050	1.7681
Creatinine-3TMS	0.05	±	0.12	0.67	±	1.28	-0.76	±	0.76	0.04	±	0.74	0.8590	0.9797	0.1940	0.9561	0.4510	1.4145
Cystathionine-4TMS	-0.27	±	0.57	1.44	±	0.61	-0.53	±	0.31	-0.64	±	0.41	0.9890	1.0110	0.0920	0.9069	0.2610	2.0010
Cysteine-3TMS	-0.52	±	0.73	-0.29	±	0.40	0.55	±	1.48	0.26	±	0.42	0.5180	0.8312	0.6010	1.1519	0.8490	0.9764
Cystine-4TMS	-0.08	±	0.59	-0.61	±	0.43	0.81	±	1.25	-0.11	±	0.82	0.1046	0.5346	0.0611	0.7665	0.6185	1.1855
Cytosine-2TMS	-1.00	±	0.06	0.81	±	0.56	-0.83	±	0.10	1.02	±	0.30	0.7306	0.9002	0.0251	0.4948	0.9526	0.9884
Decanoic-acid-TMS	0.20	±	1.26	-0.22	±	1.02	0.17	±	0.59	-0.15	±	0.82	0.8840	0.9838	0.9130	0.9921	0.8400	0.9824
Dihydroorotic-acid-3TMS	-0.81	±	1.06	0.38	±	0.77	0.03	±	0.81	0.40	±	0.68	0.5270	0.8264	0.5610	1.1555	0.7520	1.0482
Dihydrouracil-TMS	-0.29	±	1.19	-0.22	±	0.78	0.22	±	0.81	0.29	±	0.92	0.9700	1.0141	0.9630	0.9844	0.5010	1.3828
Docosahexaenoic-acid-TMS	0.44	±	1.77	-0.25	±	0.29	-0.18	±	0.24	0.00	±	0.50	0.4690	0.8746	0.2810	1.0772	0.4430	1.4217
Erythrulose-meto-3TMS(1)	-0.12	±	1.40	-0.13	±	1.07	0.23	±	0.72	0.02	±	0.31	0.3170	0.8254	0.4820	1.1878	0.3400	1.8768
Fucose-meto-4TMS(1)	-0.16	±	0.82	0.87	±	0.81	-0.72	±	0.78	0.02	±	0.75	0.0785	0.4710	0.2360	1.0178	0.4171	1.4759
Fumaric-acid-2TMS	0.43	±	1.20	0.27	±	0.66	0.00	±	1.00	-0.69	±	0.44	0.9370	0.9871	0.8890	1.0139	0.3590	1.7694
Galactosamine-5TMS(2)	-0.86	±	1.07	-0.15	±	0.60	0.33	±	0.60	0.69	±	0.77	0.3760	0.8108	0.8080	1.0722	0.7050	1.0691
Glucose6-phosphate-meto-6TMS(1)	-0.93	±	1.04	0.41	±	0.67	0.39	±	0.71	0.13	±	0.75	0.9030	0.9812	0.5440	1.2108	0.4520	1.3861
Glucose-meto-5TMS(2)	-0.50	±	1.45	-0.06	±	0.73	0.32	±	0.81	0.24	±	0.30	0.5990	0.8701	0.8870	1.0286	0.7820	1.0181
Glutamic-acid-3TMS	-1.00	±	0.63	0.03	±	0.65	0.31	±	0.98	0.66	±	0.71	0.2250	0.7962	0.6880	1.1439	0.4290	1.4440
Glutamine-3TMS	-0.82	±	1.06	0.17	±	0.62	0.16	±	0.87	0.49	±	0.75	0.2500	0.8415	0.5820	1.1640	0.6740	1.1343
Glutaric-acid-2TMS	-0.32	±	1.17	0.47	±	1.20	0.02	±	0.76	-0.18	±	0.28	0.8650	0.9784	0.2160	1.0279	0.8110	0.9993
Glycerol3-phosphate-4TMS	0.49	±	0.92	-0.34	±	0.69	0.01	±	1.27	-0.15	±	0.70	0.7040	0.8913	0.3920	1.0819	0.4190	1.4456
Glycine-3TMS	-1.05	±	0.59	-0.25	±	0.66	0.37	±	0.80	0.92	±	0.48	0.1340	0.5965	0.7940	1.0849	0.8450	0.9799
Glycolic-acid-2TMS	-0.28	±	0.84	0.43	±	1.56	0.17	±	0.39	-0.32	±	0.33	0.6140	0.8646	0.6450	1.1560	0.6870	1.1154
Glycyl-Glycine-3TMS	-0.81	±	0.95	-0.13	±	0.46	-0.11	±	0.46	1.06	±	0.81	0.2820	0.7942	0.3540	1.1361	0.5430	1.2920
Glyoxylicacid-oxime-2TMS	-0.14	±	1.06	0.11	±	1.04	-0.08	±	0.83	0.11	±	0.93	0.0442	0.5083	0.8134	1.0590	0.6386	1.1750
Guanine-3TMS	0.72	±	1.30	-1.01	±	0.35	0.40	±	0.45	-0.11	±	0.29	0.4700	0.8648	0.1190	1.0264	0.5410	1.3098
Guanosine-5TMS	-0.84	±	0.19	0.52	±	1.30	0.29	±	0.60	0.03	±	0.80	0.3770	0.8004	0.5230	1.2444	0.4900	1.4088
Hippuric-acid-TMS	-0.58	±	1.41	-0.01	±	0.77	0.26	±	0.79	0.33	±	0.31	0.6460	0.8740	0.8880	1.0212	0.6560	1.1459
Histidine-3TMS	-0.77	±	0.85	-0.61	±	0.40	0.53	±	0.83	0.86	±	0.49	0.2540	0.8346	0.9160	0.9876	0.4300	1.4129
Homoserine-2TMS	-1.09	±	0.34	0.09	±	0.77	0.37	±	1.02	0.63	±	0.59	0.0279	0.5500	0.8623	1.0720	0.9390	1.0045
Hydroxylamine-3TMS	0.02	±	1.47	-0.19	±	0.81	0.32	±	0.82	-0.16	±	0.37	0.0517	0.4459	0.3021	1.0690	0.1450	2.2233
Hypotaurine-3TMS	-0.33	±	1.17	-0.07	±	0.62	0.12	±	0.91	0.27	±	1.00	0.2220	0.8062	0.9000	1.0098	0.5280	1.3493
Hypoxanthine-2TMS	-0.79	±	0.76	-0.49	±	0.53	0.65	±	0.97	0.62	±	0.60	0.7140	0.8877	0.7770	1.1169	0.3790	1.5383

Supplementary Table 1. (Continued)

Inosine-4TMS	-0.65	±	0.78	-0.01	±	0.48	-0.48	±	0.72	1.15	±	0.68	0.0306	0.5279	0.5961	1.1586		0.7392	1.0626
Inositol-6TMS(2)	-0.75	±	0.75	0.02	±	0.85	-0.20	±	0.77	0.94	±	0.67	0.6960	0.8976	0.8500	1.0664		0.3440	1.8258
Isocitric-acid-4TMS	0.83	±	1.09	0.11	±	0.38	-0.01	±	0.53	-0.92	±	0.80	0.6540	0.8678	0.4120	1.1148		0.5710	1.3133
Isoleucine-TMS	-0.23	±	1.09	-0.43	±	0.51	0.42	±	1.08	0.25	±	0.84	0.1480	0.6189	0.8110	1.0659		0.9400	0.9902
Lactic-acid-2TMS	-0.89	±	0.63	-0.31	±	0.57	0.88	±	1.00	0.32	±	0.55	0.3570	0.8076	0.4210	1.0962		0.6600	1.1244
Lauric-acid-TMS	0.13	±	1.42	-0.08	±	0.95	0.06	±	0.78	-0.11	±	0.50	0.7000	0.8944	0.5050	1.2226		0.5920	1.2968
Leucine-2TMS	-0.89	±	0.61	0.04	±	0.82	0.33	±	0.95	0.52	±	0.83	0.0468	0.4968	0.7785	1.1076		0.7907	0.9920
Lysine-4TMS	-0.97	±	0.85	-0.34	±	0.63	0.47	±	0.69	0.84	±	0.44	0.4850	0.8366	0.6090	1.1357		0.8130	0.9929
Maleic-acid-2TMS	-0.91	±	0.27	0.13	±	0.30	0.25	±	1.15	0.53	±	1.05	0.0372	0.5134	0.5852	1.1537		0.5330	1.3135
Malic-acid-3TMS	-0.26	±	0.97	0.49	±	0.33	-0.33	±	1.30	0.10	±	0.78	0.3540	0.8142	0.3020	1.0967		0.3740	1.7204
Maltitol-9TMS	-0.25	±	1.55	-0.09	±	0.84	0.14	±	0.57	0.21	±	0.46	0.5700	0.8644	0.6490	1.1482		0.7410	1.0542
Mannitol-6TMS	-0.50	±	1.44	-0.05	±	0.76	0.31	±	0.82	0.24	±	0.29	0.6340	0.8663	0.8430	1.0772		0.7910	0.9834
Mannose6-phosphate-meto-6TMS(2)	-0.84	±	1.03	0.44	±	0.88	0.20	±	0.62	0.20	±	0.78	0.8870	0.9792	0.6850	1.1528		0.3740	1.6129
Mannose-meto-5TMS(1)	-0.41	±	1.55	-0.15	±	0.78	0.29	±	0.61	0.27	±	0.29	0.9700	1.0065	0.7280	1.1040		0.7880	1.0163
Margaric-acid-TMS	0.01	±	1.31	-0.46	±	0.46	0.28	±	0.78	0.17	±	0.96	0.2090	0.8012	0.3910	1.1012		0.5850	1.3021
meso-Erythritol-4TMS	-0.77	±	0.34	0.93	±	0.45	-1.05	±	0.09	0.89	±	0.39	0.1453	0.6267	0.0011	0.0745	*	0.2785	2.0229
Methionine-2TMS	-1.08	±	0.51	-0.26	±	0.66	0.62	±	0.89	0.72	±	0.43	0.0497	0.4572	0.7209	1.1054		0.7742	1.0273
Methylmalonic-acid-2TMS	-0.96	±	0.49	0.01	±	0.85	0.30	±	0.90	0.65	±	0.77	0.0945	0.5216	0.8868	1.0371		0.6144	1.2288
Monostearin-2TMS	-0.01	±	1.56	0.05	±	0.86	0.05	±	0.51	-0.10	±	0.59	0.3310	0.8157	0.9560	0.9919		0.6860	1.1270
Myristic-acid-TMS	-0.04	±	1.33	-0.42	±	0.60	0.39	±	0.93	0.07	±	0.67	0.3850	0.7813	0.3570	1.1197		0.6080	1.2713
N6-Acetyllysine-2TMS	-0.89	±	0.44	0.12	±	0.70	0.03	±	0.90	0.73	±	0.99	0.2030	0.8004	0.5470	1.1982		0.4750	1.3947
N-Acetylaspartic-acid-3TMS	-0.26	±	0.92	0.58	±	0.75	0.09	±	1.04	-0.41	±	0.85	0.3396	0.8222	0.0818	0.8683		0.6837	1.1368
N-Acetylglutamine-3TMS	-0.94	±	1.00	-0.11	±	0.77	0.43	±	0.66	0.62	±	0.56	0.2740	0.8220	0.3140	1.0569		0.9400	0.9978
N-Acetylneuraminic-acid-6TMS	-0.12	±	1.12	-0.01	±	1.18	-0.16	±	0.68	0.30	±	0.76	0.5975	0.8772	0.5345	1.2502		0.0691	1.9072
N-Acetylserine-2TMS	0.52	±	1.81	-0.33	±	0.10	-0.21	±	0.15	0.02	±	0.25	0.5790	0.8592	0.1740	0.9235		0.6250	1.1815
N-Butyrylglycine-TMS	-0.16	±	0.79	-0.40	±	1.12	0.41	±	1.24	0.15	±	0.09	0.9890	1.0035	0.5550	1.1967		0.9280	1.0084
Niacinamide-TMS	-0.92	±	0.75	-0.26	±	0.67	0.48	±	0.72	0.70	±	0.80	0.9971	0.9971	0.8765	1.0427		0.0922	2.1206
Nonanoic-acid-TMS	-0.23	±	0.80	0.06	±	1.08	0.49	±	0.75	-0.33	±	1.02	0.1040	0.5520	0.8050	1.0785		0.2500	2.0294
Octadecanol-TMS	-0.20	±	1.15	-0.48	±	0.38	0.35	±	0.82	0.33	±	1.07	0.0842	0.4842	0.6836	1.1647		0.3701	1.7612
Octanoic-acid-TMS	0.14	±	1.66	-0.07	±	0.49	0.30	±	0.44	-0.37	±	0.58	0.5130	0.8329	0.3680	1.1040		0.3780	1.5807
Oleamide-TMS	0.58	±	1.59	-0.46	±	0.39	-0.42	±	0.17	0.30	±	0.53	0.6241	0.8700	0.0703	0.8085		0.2014	1.8529
Oleic-acid-TMS	0.73	±	1.74	-0.30	±	0.04	-0.26	±	0.13	-0.16	±	0.19	0.6120	0.8707	0.1330	1.0197		0.5290	1.3273
O-Phosphoethanolamine-4TMS	-0.13	±	1.29	-0.27	±	0.60	-0.08	±	0.79	0.48	±	0.91	0.3300	0.8280	0.8920	1.0090		0.7890	1.0082
Ornithine-4TMS	-0.94	±	0.40	-0.73	±	0.41	0.65	±	0.58	1.03	±	0.49	0.0751	0.4935	0.9515	1.0023		0.9419	0.9847
Oxalic-acid-2TMS	0.39	±	1.42	-0.31	±	0.65	0.23	±	0.74	-0.31	±	0.64	0.3230	0.8254	0.7330	1.0995		0.6150	1.2124

Supplementary Table 1. (Continued)

Palmitic-acid-TMS	0.42	±	1.73	-0.31	±	0.40	-0.08	±	0.29	-0.03	±	0.53	0.4170	0.8105	0.3010	1.1226		0.9620	0.9907
Palmitoleic-acid-TMS	0.41	±	1.69	-0.53	±	0.25	0.03	±	0.39	0.08	±	0.52	0.5450	0.8357	0.1520	0.9535		0.7090	1.0635
Pantothenic-acid-3TMS	-0.80	±	0.13	0.66	±	1.10	-0.75	±	0.27	0.89	±	0.28	0.4820	0.8528	0.9580	0.9866		0.6060	1.2866
Phenylalanine-2TMS	-0.94	±	0.66	-0.37	±	0.68	0.62	±	0.89	0.70	±	0.46	0.2650	0.8311	0.7960	1.0769		0.7010	1.0869
Phenylpyruvic-acid-meto-TMS(1)	0.16	±	1.58	-0.20	±	0.70	0.25	±	0.25	-0.21	±	0.75	0.7880	0.9294	0.7510	1.1025		0.4560	1.3680
Phosphoric-acid-3TMS	-0.59	±	0.67	-0.22	±	0.61	0.67	±	1.35	0.14	±	0.54	0.2540	0.8152	0.4390	1.1219		0.7050	1.0810
Proline-2TMS	-0.90	±	0.42	0.01	±	0.52	0.14	±	0.96	0.75	±	1.02	0.0381	0.4780	0.6930	1.1385		0.9232	1.0111
Psicose-meto-5TMS(2)	-0.47	±	0.96	0.12	±	0.90	0.66	±	1.02	-0.31	±	0.50	0.9080	0.9789	0.4530	1.1366		0.5570	1.3028
Pyridoxal-meto-2TMS(1)	-0.25	±	1.16	0.24	±	1.09	0.10	±	0.54	-0.09	±	0.92	0.3440	0.8185	0.7140	1.1071		0.6100	1.2564
Pyridoxine-3TMS	-0.45	±	1.34	-0.08	±	0.37	0.77	±	0.92	-0.23	±	0.42	0.6560	0.8622	0.3050	1.0523		0.6510	1.1518
Pyrogallol-3TMS	0.08	±	0.51	-0.16	±	0.54	0.48	±	1.65	-0.39	±	0.36	0.0185	0.5106	0.9228	0.9872		0.0926	1.8255
Pyruvic-acid-meto-TMS	0.09	±	0.49	0.77	±	1.37	-0.28	±	0.78	-0.57	±	0.27	0.9340	0.9915	0.1680	0.9660		0.8780	0.9931
Rhamnose-meto-4TMS(1)	-0.29	±	0.52	0.91	±	1.17	-0.56	±	0.66	-0.06	±	0.70	0.9020	0.9879	0.9840	0.9912		0.2890	1.8128
Ribonic-acid-5TMS	-0.82	±	0.47	-0.82	±	0.33	0.90	±	0.74	0.74	±	0.47	0.3900	0.7689	0.6380	1.1585		0.3740	1.6649
Ribulose-meto-4TMS	-0.39	±	0.77	-0.31	±	0.63	-0.04	±	0.95	0.74	±	1.05	0.3880	0.7760	0.8150	1.0511		0.6000	1.2938
Sebacic-acid-2TMS	-0.77	±	0.72	-0.52	±	0.74	0.21	±	0.28	1.08	±	0.77	0.3840	0.8029	0.9010	1.0027		0.5270	1.3986
Serine-3TMS	-1.09	±	0.42	0.04	±	0.74	0.39	±	0.98	0.65	±	0.58	0.0358	0.5489	0.7922	1.0932		0.9359	1.0090
Stearic-acid-TMS	0.18	±	1.57	-0.17	±	0.73	0.03	±	0.47	-0.04	±	0.71	0.3060	0.8280	0.6730	1.1756		0.7370	1.0706
Succinic-acid-2TMS	0.28	±	1.83	-0.30	±	0.25	0.02	±	0.36	0.00	±	0.29	0.2190	0.8168	0.2330	1.0718		0.6880	1.1040
Sucrose-8TMS	0.88	±	0.88	-0.82	±	0.11	0.73	±	0.53	-0.80	±	0.34	0.6869	0.8943	0.0191	0.4393		0.6591	1.1369
Tagatose-meto-5TMS(2)	-0.26	±	1.24	-0.08	±	0.64	0.70	±	0.82	-0.36	±	0.69	0.6300	0.8694	0.3900	1.1451		0.1460	2.0148
Taurine-3TMS	-0.85	±	1.11	-0.32	±	0.39	0.54	±	0.77	0.63	±	0.56	0.8440	0.9706	0.8660	1.0576		0.9030	1.0050
Threitol-4TMS	-0.84	±	0.27	0.89	±	0.33	-1.02	±	0.13	0.97	±	0.37	0.2760	0.7935	0.0006	0.0822	*	0.2860	1.9734
Threo-b-hydroxyaspartic-acid-4TMS	0.11	±	0.65	-0.30	±	0.61	0.33	±	1.65	-0.15	±	0.22	0.1087	0.5357	0.3910	1.1241		0.0443	1.5284
Threonine-3TMS	-1.11	±	0.55	0.01	±	0.76	0.39	±	0.89	0.71	±	0.50	0.0199	0.4577	0.7040	1.1040		0.7531	1.0393
Tryptamine-2TMS	-0.81	±	0.17	0.68	±	1.08	-0.74	±	0.32	0.87	±	0.23	0.4300	0.8242	0.9040	0.9980		0.8320	0.9984
Tryptophan-3TMS	0.41	±	1.72	-0.31	±	0.40	-0.23	±	0.44	0.14	±	0.39	0.6520	0.8736	0.2560	1.0705		0.3890	1.4127
Tyrosine-3TMS	-0.90	±	0.97	-0.09	±	0.81	0.27	±	0.78	0.72	±	0.43	0.5060	0.8313	0.6780	1.1696		0.8310	1.0059
Uracil-2TMS	0.60	±	1.47	-0.09	±	0.79	-0.15	±	0.67	-0.37	±	0.16	0.4840	0.8455	0.1740	0.9605		0.7510	1.0575
Urea-2TMS	0.47	±	0.22	-0.78	±	0.56	0.81	±	1.24	-0.50	±	0.42	0.0052	0.7107	0.0018	0.0846	*	0.0174	1.2034
Ureidopropionic-acid-3TMS	-0.19	±	1.40	-0.45	±	0.33	0.62	±	0.92	0.02	±	0.52	0.5000	0.8313	0.4160	1.1040		0.2910	1.7460
Ureidosuccinic-acid-3TMS	-0.12	±	1.44	-0.20	±	0.70	0.10	±	0.97	0.21	±	0.45	0.8810	0.9884	0.5440	1.2307		0.6890	1.0929
Uric-acid-4TMS	0.28	±	0.31	1.24	±	0.54	-1.04	±	0.48	-0.48	±	0.48	0.4860	0.8280	0.5560	1.1804		0.6930	1.0868
Valine-2TMS	-1.00	±	0.44	-0.05	±	0.77	0.25	±	0.81	0.80	±	0.79	0.0550	0.4217	0.8730	1.0568		0.9700	0.9843
Xanthine-3TMS	-0.85	±	0.60	0.58	±	1.20	-0.29	±	0.63	0.56	±	0.38	0.7950	0.9297	0.8640	1.0646		0.1480	1.8567

Supplementary Table 1. (Continued)

Xylulose-meto-4TMS	-0.84	±	0.59	-0.62	±	0.53	0.43	±	0.78	1.03	±	0.49	0.2260	0.7797	0.2670	1.0527	0.9710	0.9781

Supplementary Table 2. All albumen metabolites.

Albumen metabolite	Relative area (mean ± SD)				Two-way mixed design ANOVA						
	RIR	RIR	AUS	AUS	Breed		Feed			Breed*Feed	
	Mixed	Fermented	Mixed	Fermented	p-value	q-value	p-value	q-value		p-value	q-value
1-Hexadecanol-TMS	-0.12 ± 1.22	-0.01 ± 0.65	-0.35 ± 0.42	0.48 ± 1.28	0.8490	0.9745	0.3630	0.7606		0.3940	0.9124
2-3-Bisphosphoglyceric-acid-5TMS	0.32 ± 0.57	0.64 ± 1.28	-0.58 ± 0.68	-0.38 ± 0.68	0.0458	0.5038	0.6967	0.9289		0.8673	1.0131
2-Aminobutyric-acid-2TMS	0.55 ± 1.64	-0.12 ± 0.45	-0.36 ± 0.54	-0.07 ± 0.85	0.1970	0.6501	0.2020	0.5673		0.2950	1.0524
2-Aminoethanol-2TMS	0.79 ± 0.98	-0.58 ± 0.74	0.11 ± 1.10	-0.33 ± 0.44	0.0165	0.7260	0.8107	0.9641		0.2376	1.0454
2-Aminoisobutyric-acid-2TMS	0.64 ± 1.38	0.17 ± 0.92	-0.53 ± 0.42	-0.29 ± 0.67	0.1330	0.6270	0.3730	0.7575		0.5480	0.9156
2-Aminopimelic-acid-3TMS	0.58 ± 1.03	-0.20 ± 0.99	-0.15 ± 1.11	-0.23 ± 0.53	0.9660	0.9962	0.7410	0.9141		0.1130	1.1474
2-Deoxy-glucose-4TMS(2)	0.10 ± 1.43	0.34 ± 0.91	-0.42 ± 0.63	-0.02 ± 0.80	0.2110	0.6631	0.5280	0.8297		0.5160	0.9871
2-Hydroxyglutaric-acid-3TMS	0.09 ± 1.01	-0.89 ± 0.56	0.64 ± 0.46	0.16 ± 1.11	0.8490	0.9661	0.9440	1.0049		0.4760	0.9378
2-Ketobutyric-acid-meto-TMS(2)	0.40 ± 0.92	0.18 ± 1.16	-0.35 ± 0.52	-0.23 ± 1.06	0.6080	0.8448	0.2960	0.6977		0.7380	0.9840
2-Ketoisocaproic-acid-meto-TMS(1)	0.45 ± 0.88	-0.38 ± 0.43	0.03 ± 1.25	-0.10 ± 1.04	0.4773	0.8077	0.3778	0.7334		0.0485	0.7113
2-Propyl-5-hydroxy-pentanoic-acid-2TMS	0.17 ± 1.19	0.07 ± 0.40	-0.37 ± 1.37	0.13 ± 0.66	0.3910	0.8602	0.8620	0.9725		0.4170	0.9024
3-Aminopropanoic-acid-3TMS	0.78 ± 1.25	0.35 ± 0.94	-0.50 ± 0.44	-0.63 ± 0.32	0.4160	0.8716	0.2570	0.6652		0.5180	0.9768
3-Hydroxyanthranilic-acid-3TMS	0.23 ± 1.06	0.23 ± 0.98	-0.47 ± 0.87	0.02 ± 0.92	0.0450	0.5400	0.4120	0.7553		0.5680	0.9372
3-Hydroxybutyric-acid-2TMS	0.71 ± 0.70	0.18 ± 1.38	-0.55 ± 0.71	-0.34 ± 0.22	0.2785	0.7502	0.0695	0.2867		0.0235	1.0340
3-Hydroxyglutaric-acid-3TMS	0.18 ± 0.58	-0.18 ± 0.69	0.27 ± 1.41	-0.26 ± 0.93	0.2220	0.6512	0.9190	0.9943		0.1110	1.2210
3-Hydroxyisobutyric-acid-2TMS	0.79 ± 0.81	0.06 ± 1.25	-0.64 ± 0.71	-0.21 ± 0.35	0.3760	0.8412	0.1032	0.3682		0.0123	0.8118
3-Hydroxyisovaleric-acid-2TMS	0.46 ± 1.13	-0.59 ± 0.65	0.10 ± 0.67	0.03 ± 1.15	0.9840	0.9915	0.8460	0.9711		0.1610	1.0120
3-Methyl-2-oxovaleric-acid-meto-TMS(1)	0.42 ± 1.75	0.16 ± 0.46	-0.39 ± 0.63	-0.20 ± 0.59	0.1080	0.5702	0.0950	0.3483		0.6250	0.9706
4-Hydroxyphenyllactic-acid-3TMS	0.41 ± 0.79	-1.05 ± 0.52	1.05 ± 0.43	-0.40 ± 0.57	0.8586	0.9604	0.0069	0.0907	*	0.3421	0.9609
5-Dehydroquinic-acid-5TMS	0.46 ± 1.26	0.36 ± 0.90	-0.57 ± 0.90	-0.25 ± 0.42	0.4330	0.8531	0.8670	0.9699		0.2240	1.0951
5-Oxoproline-2TMS	0.55 ± 1.14	-0.76 ± 0.74	0.75 ± 0.52	-0.55 ± 0.46	0.5760	0.8448	0.0218	0.1693		0.7883	1.0303
Adenine-2TMS	0.33 ± 1.34	0.50 ± 0.91	-0.55 ± 0.67	-0.28 ± 0.59	0.2790	0.7366	0.5430	0.8239		0.2510	0.9466
Adenosine-4TMS	0.48 ± 1.35	-0.49 ± 0.82	0.61 ± 0.64	-0.60 ± 0.23	0.3550	0.8221	0.4630	0.8042		0.5430	0.9309
Alanine-2TMS	0.54 ± 1.29	-0.75 ± 0.39	0.85 ± 0.55	-0.64 ± 0.18	0.4580	0.7955	0.0075	0.0822	*	0.9401	1.0089
Arabitol-5TMS	-0.24 ± 0.66	0.61 ± 0.54	-0.44 ± 1.51	0.07 ± 0.49	0.0350	0.6600	0.0499	0.2533		0.0259	0.6838
Arachidonic-acid-TMS	0.34 ± 1.09	0.09 ± 1.20	-0.57 ± 0.83	0.14 ± 0.49	0.9120	0.9868	0.7220	0.9253		0.8560	1.0272
Arginine-3TMS	0.51 ± 1.56	0.15 ± 0.53	-0.55 ± 0.33	-0.11 ± 0.94	0.8700	0.9650	0.3210	0.7306		0.1500	1.0421
Ascorbic-acid-4TMS	0.58 ± 1.47	0.47 ± 0.59	-0.67 ± 0.49	-0.37 ± 0.59	0.8410	0.9738	0.4970	0.8099		0.4120	0.9064
Aspartic-acid-3TMS	0.43 ± 1.46	-0.54 ± 0.33	0.65 ± 0.89	-0.54 ± 0.09	0.7382	0.9553	0.0451	0.2481		0.9471	0.9922
Benzoic-acid-TMS	1.14 ± 0.70	-0.05 ± 0.97	-0.31 ± 0.32	-0.79 ± 0.58	0.3530	0.8321	0.2140	0.5885		0.3180	0.9762
Cadaverine-4TMS	0.86 ± 1.49	-0.05 ± 0.80	-0.34 ± 0.28	-0.48 ± 0.45	0.1040	0.5720	0.5360	0.8324		0.8990	1.0057
Caproic-acid-TMS	0.76 ± 1.42	-0.03 ± 0.89	-0.26 ± 0.45	-0.46 ± 0.58	0.5280	0.8200	0.8870	0.9839		0.1920	1.1019

Supplementary Table 2. (Continued)

Citric-acid-4TMS	0.50 ± 1.10	-0.11 ± 1.08	-0.19 ± 1.05	-0.20 ± 0.49	0.9800	0.9951	0.7980	0.9664		0.1640	0.9840
Cysteic-acid-3TMS	-0.11 ± 0.75	-0.25 ± 0.68	0.79 ± 1.40	-0.43 ± 0.22	0.6820	0.9093	0.7050	0.9306		0.2180	1.1068
Cysteine-3TMS	0.33 ± 1.51	0.05 ± 0.78	-0.01 ± 0.86	-0.38 ± 0.60	0.9260	0.9857	0.8050	0.9660		0.5370	0.9451
Decanoic-acid-TMS	1.13 ± 1.11	-0.44 ± 0.51	-0.27 ± 0.48	-0.43 ± 0.76	0.0850	0.5610	0.4530	0.8081		0.6910	0.9403
Dihydrouracil-TMS	-0.57 ± 0.93	0.69 ± 0.93	-0.77 ± 0.48	0.65 ± 0.43	0.9300	0.9821	0.0000	0.0013	*	0.8510	1.0306
Dihydroxyacetone-phosphate-meto-3TMS(1)	0.10 ± 1.45	-0.23 ± 0.76	0.07 ± 0.97	0.06 ± 0.72	0.3360	0.8368	0.9250	0.9927		0.2770	1.0157
Docosahexaenoic-acid-TMS	0.70 ± 1.28	0.01 ± 0.71	-0.76 ± 0.79	0.04 ± 0.54	0.5840	0.8379	0.9550	1.0005		0.7970	1.0314
Elaidic-acid-TMS	0.47 ± 1.15	0.23 ± 1.11	-0.73 ± 0.42	0.03 ± 0.71	0.0350	0.5775	0.1990	0.5710		0.6600	0.9368
Erythrose-4-phosphate-meto-4TMS(1)	0.35 ± 1.38	-0.15 ± 1.05	-0.01 ± 0.78	-0.20 ± 0.63	0.8370	0.9865	0.0934	0.3523		0.2358	1.0733
Erythrulose-meto-3TMS(1)	1.01 ± 1.40	-0.19 ± 0.45	-0.42 ± 0.47	-0.40 ± 0.64	0.8350	0.9930	0.5750	0.8433		0.1470	1.0780
Fucose-meto-4TMS(1)	0.45 ± 0.58	0.38 ± 1.39	-0.74 ± 0.73	-0.09 ± 0.40	0.5490	0.8427	0.4800	0.8020		0.1320	1.1616
Fumaric-acid-2TMS	-0.45 ± 1.32	-0.51 ± 0.63	0.71 ± 0.58	0.25 ± 0.81	0.9060	0.9884	0.9580	0.9957		0.8040	1.0205
Gluconic-acid-6TMS	0.97 ± 1.76	-0.45 ± 0.09	-0.16 ± 0.10	-0.35 ± 0.12	0.4611	0.7905	0.0893	0.3467		0.5416	0.9407
Glucose-6-phosphate-meto-6TMS(1)	-0.07 ± 1.02	-0.42 ± 0.97	1.01 ± 0.72	-0.52 ± 0.21	0.9520	0.9973	0.0680	0.2895		0.7290	0.9819
Glutamic-acid-3TMS	0.48 ± 1.53	-0.59 ± 0.40	0.66 ± 0.64	-0.55 ± 0.17	0.7836	0.9667	0.0179	0.1477		0.8388	1.0252
Glutamine-3TMS	0.05 ± 0.90	-0.49 ± 0.71	0.86 ± 1.14	-0.42 ± 0.44	0.3644	0.8293	0.0516	0.2523		0.8102	1.0185
Glutaric-acid-2TMS	0.37 ± 0.88	0.08 ± 1.01	-0.74 ± 0.52	0.30 ± 1.06	0.4540	0.8098	0.1810	0.5556		0.7980	1.0227
Glyceric-acid-3TMS	0.09 ± 1.15	-0.12 ± 1.33	0.12 ± 0.78	-0.09 ± 0.57	0.2810	0.7273	0.1570	0.4934		0.3130	0.9837
Glycerol-3-phosphate-4TMS	0.04 ± 1.29	-0.25 ± 0.94	0.02 ± 0.83	0.19 ± 0.88	0.4450	0.8638	0.9090	0.9916		0.2470	0.9880
Glycerol-3TMS	0.87 ± 1.10	0.40 ± 0.81	-0.42 ± 0.40	-0.86 ± 0.42	0.2240	0.6428	0.8340	0.9742		0.2980	1.0352
Glycine-3TMS	0.44 ± 1.28	-0.70 ± 0.73	0.83 ± 0.42	-0.57 ± 0.25	0.8517	0.9608	0.0035	0.0664	*	0.9328	1.0176
Glycolic-acid-2TMS	-0.48 ± 0.44	0.89 ± 1.53	-0.46 ± 0.17	0.04 ± 0.24	0.9870	0.9870	0.5660	0.8490		0.6810	0.9364
Glyoxylic-acid-oxime-2TMS	0.52 ± 1.35	-0.48 ± 0.66	-0.03 ± 0.69	-0.01 ± 0.96	0.1530	0.6311	0.4520	0.8173		0.3580	0.9451
Guanine-3TMS	0.96 ± 1.49	0.03 ± 0.62	-0.50 ± 0.29	-0.49 ± 0.36	0.4960	0.7984	0.8250	0.9723		0.9530	0.9905
Guanosine-5TMS	-0.31 ± 0.72	-0.39 ± 1.22	0.08 ± 0.68	0.61 ± 0.90	0.4510	0.8385	0.3730	0.7460		0.3920	0.9240
Hippuric-acid-TMS	0.69 ± 1.10	0.37 ± 1.06	-0.53 ± 0.56	-0.53 ± 0.46	0.4010	0.8677	0.5700	0.8454		0.3190	0.9570
Histidine-3TMS	0.13 ± 1.07	-0.58 ± 0.33	0.97 ± 1.07	-0.52 ± 0.24	0.7790	0.9701	0.2190	0.5900		0.7680	1.0138
Hydroxylamine-3TMS	0.85 ± 1.09	0.05 ± 0.63	-0.10 ± 0.37	-0.79 ± 0.98	0.8057	0.9757	0.4877	0.8047		0.0469	0.7739
Hypoxanthine-2TMS	-0.52 ± 1.03	-0.62 ± 0.94	0.53 ± 0.57	0.62 ± 0.65	0.2260	0.6347	0.5410	0.8304		0.4720	0.9440
Inosine-4TMS	-0.53 ± 0.94	-0.30 ± 1.04	0.52 ± 0.79	0.31 ± 0.81	0.8400	0.9812	0.6410	0.9197		0.3820	0.9697
Inositol-6TMS(2)	0.79 ± 0.74	0.37 ± 0.85	-0.81 ± 0.89	-0.35 ± 0.54	0.0549	0.4831	0.7281	0.9153		0.3882	0.9317
Isocitric-acid-4TMS	0.82 ± 0.84	-0.60 ± 0.71	-0.06 ± 1.23	-0.16 ± 0.38	0.8110	0.9732	0.6790	0.9336		0.1360	1.0560
Isoleucine-TMS	0.26 ± 0.77	-0.89 ± 0.63	1.20 ± 0.41	-0.57 ± 0.39	0.4861	0.8020	0.0008	0.0199	*	0.6411	0.9508
Lactic-acid-2TMS	0.92 ± 1.01	-0.42 ± 0.53	0.29 ± 0.95	-0.79 ± 0.23	0.0339	0.7458	0.5179	0.8337		0.0405	0.8910
Lauric-acid-TMS	0.61 ± 0.96	-0.06 ± 0.68	-0.63 ± 0.79	0.09 ± 1.10	0.1690	0.6197	0.8600	0.9786		0.6460	0.9371

Supplementary Table 2. (Continued)

Leucine-2TMS	0.43 ± 1.42	-0.69 ± 0.23	0.86 ± 0.61	-0.60 ± 0.06	0.6514	0.8774	0.0829	0.3316		0.9365	1.0133	
Linoleic-acid-TMS	-0.02 ± 0.88	0.81 ± 1.34	-0.75 ± 0.32	-0.04 ± 0.25	0.0488	0.4957	0.0002	0.0082	*	0.4784	0.9287	
Lysine-4TMS	0.23 ± 1.23	-0.62 ± 0.71	0.90 ± 0.61	-0.51 ± 0.44	0.4530	0.8305	0.1070	0.3622		0.4400	0.8935	
Malic-acid-3TMS	0.47 ± 1.59	-0.39 ± 0.72	0.31 ± 0.58	-0.39 ± 0.56	0.9240	0.9916	0.1080	0.3564		0.4100	0.9173	
Maltose-meto-8TMS(1)	0.78 ± 0.61	0.65 ± 1.13	-0.66 ± 0.32	-0.77 ± 0.29	0.1479	0.6298	0.0366	0.2416		0.3072	1.0138	
Mannitol-6TMS	0.56 ± 1.10	0.34 ± 1.16	-0.43 ± 0.61	-0.47 ± 0.51	0.4230	0.8590	0.3440	0.7696		0.2340	1.1031	
Mannose-meto-5TMS(1)	0.92 ± 1.24	0.23 ± 0.76	-0.52 ± 0.28	-0.62 ± 0.61	0.7870	0.9619	0.9610	0.9910		0.3740	0.9680	
Margaric-acid-TMS	0.18 ± 0.99	0.18 ± 0.52	-0.61 ± 0.85	0.25 ± 1.23	0.3460	0.8304	0.3450	0.7590		0.8610	1.0239	
meso-Erythritol-4TMS	-0.67 ± 0.20	1.13 ± 0.72	-1.04 ± 0.12	0.58 ± 0.30	0.3389	0.8284	0.0005	0.0173	*	0.9123	1.0035	
Methionine-2TMS	0.09 ± 1.31	-0.68 ± 0.34	0.97 ± 0.83	-0.38 ± 0.23	0.6266	0.8616	0.0675	0.2970		0.8383	1.0342	
Monostearin-2TMS	0.34 ± 1.06	-0.14 ± 0.47	-0.77 ± 0.48	0.58 ± 1.18	0.0077	0.5069	0.6821	0.9282		0.1243	1.1718	
Myristic-acid-TMS	0.10 ± 0.87	0.17 ± 1.14	-0.56 ± 0.86	0.29 ± 0.85	0.0831	0.5773	0.2956	0.7094		0.4384	0.9042	
N-Acetylaspartic-acid-2TMS	-0.68 ± 0.54	-0.88 ± 0.21	0.50 ± 0.67	1.06 ± 0.69	0.7200	0.9410	0.2240	0.5914		0.6540	0.9383	
N-Acetylglutamine-3TMS	0.50 ± 1.09	0.40 ± 1.27	-0.43 ± 0.52	-0.47 ± 0.40	0.4850	0.8104	0.2930	0.7297		0.3280	0.9412	
N-Acetyl-Lysine-2TMS	0.39 ± 1.06	0.46 ± 1.27	-0.41 ± 0.59	-0.43 ± 0.45	0.4530	0.8191	0.3940	0.7430		0.2400	1.0219	
N-Acetylmannosamine-meto-4TMS(1)	0.36 ± 0.87	0.26 ± 1.07	-0.51 ± 0.90	-0.10 ± 0.89	0.0424	0.5597	0.9690	0.9915		0.6435	0.9438	
N-Butyrylglycine-TMS	0.12 ± 0.78	-0.34 ± 0.72	-0.04 ± 1.02	0.26 ± 1.24	0.3213	0.8156	0.0001	0.0069	*	0.0000	0.0048	*
Niacinamide-TMS	-0.21 ± 1.22	-0.15 ± 0.78	0.26 ± 1.06	0.10 ± 0.86	0.4195	0.8652	0.7212	0.9426		0.0424	0.7995	
Nonanoic-acid-TMS	0.96 ± 1.32	-0.18 ± 0.50	-0.18 ± 0.76	-0.60 ± 0.48	0.1810	0.6287	0.6180	0.8964		0.9750	0.9977	
Octadecanol-TMS	0.03 ± 0.70	0.23 ± 0.75	-0.70 ± 0.56	0.45 ± 1.34	0.1610	0.6251	0.4570	0.8043		0.5350	0.9674	
Octanoic-acid-TMS	0.82 ± 1.41	-0.59 ± 0.87	-0.06 ± 0.48	-0.17 ± 0.43	0.4460	0.8532	0.4770	0.8072		0.2990	1.0120	
Oleamide-TMS	0.07 ± 1.12	-0.15 ± 1.36	-0.18 ± 0.63	0.26 ± 0.65	0.4930	0.8034	0.6460	0.9169		0.8660	1.0206	
Oleic-acid-TMS	0.34 ± 1.16	0.46 ± 1.20	-0.53 ± 0.34	-0.27 ± 0.70	0.2155	0.6465	0.0397	0.2278		0.9952	1.0028	
O-Phosphoethanolamine-4TMS	-0.21 ± 0.48	0.95 ± 1.33	-0.74 ± 0.17	0.01 ± 0.54	0.7610	0.9753	0.4680	0.8023		0.6600	0.9268	
Ornithine-3TMS	0.37 ± 0.93	-0.63 ± 0.91	0.66 ± 0.70	-0.41 ± 0.82	0.0710	0.5513	0.0452	0.2387		0.9088	1.0081	
Oxalic-acid-2TMS	0.51 ± 1.38	0.16 ± 0.96	-0.48 ± 0.59	-0.19 ± 0.68	0.0912	0.5733	0.2938	0.7182		0.5811	0.9354	
Palmitic-acid-TMS	0.33 ± 1.28	0.29 ± 0.90	-0.79 ± 0.44	0.18 ± 0.81	0.0786	0.5764	0.1900	0.5573		0.3248	0.9527	
Palmitoleic-acid-TMS	0.40 ± 1.52	-0.14 ± 0.43	-0.85 ± 0.50	0.59 ± 0.51	0.5720	0.8580	0.9990	1.0066		0.9660	0.9962	
Pantothenic-acid-3TMS	0.05 ± 0.44	0.53 ± 1.59	-0.67 ± 0.24	0.09 ± 0.56	0.9574	0.9951	0.0662	0.3013		0.8839	1.0058	
Phenylacetic-acid-TMS	-0.68 ± 0.32	1.43 ± 0.56	-0.90 ± 0.08	0.15 ± 0.21	0.2408	0.6622	0.0366	0.2301		0.6387	0.9691	
Phenylalanine-2TMS	-0.12 ± 1.26	-0.57 ± 0.53	0.62 ± 1.05	0.06 ± 0.64	0.4250	0.8500	0.8350	0.9668		0.6380	0.9793	
Phenylpyruvic-acid-meto-TMS(1)	0.91 ± 1.41	0.07 ± 0.50	-0.78 ± 0.66	-0.20 ± 0.23	0.1830	0.6194	0.3620	0.7707		0.6000	0.9429	
Phosphoric-acid-3TMS	0.65 ± 0.99	0.13 ± 1.07	-0.56 ± 0.92	-0.22 ± 0.47	0.1030	0.5911	1.0000	1.0000		0.1320	1.0890	
Proline-2TMS	0.45 ± 1.42	-0.66 ± 0.33	0.83 ± 0.58	-0.63 ± 0.13	0.4981	0.7922	0.0306	0.2244		0.8920	1.0064	
Psicose-meto-5TMS(1)	0.79 ± 1.46	0.09 ± 0.84	-0.27 ± 0.37	-0.60 ± 0.43	0.1390	0.6116	0.6960	0.9375		0.4320	0.9051	

Supplementary Table 2. (Continued)

Pyridoxamine-3TMS	0.40 ± 1.10	-0.92 ± 0.60	0.97 ± 0.43	-0.46 ± 0.35	0.9730	0.9956		0.0163	0.1537		0.9984	0.9984
Pyridoxine-3TMS	0.54 ± 0.85	0.55 ± 1.20	-0.52 ± 0.59	-0.57 ± 0.48	0.4570	0.8043		0.3960	0.7362		0.3860	0.9436
Pyruvic-acid-meto-TMS	-0.06 ± 1.36	0.19 ± 0.99	-0.17 ± 1.07	0.04 ± 0.39	0.1551	0.6202		0.0050	0.0832	*	0.3117	1.0037
Rhamnose-meto-4TMS(1)	0.41 ± 0.43	0.81 ± 1.16	-0.70 ± 0.67	-0.52 ± 0.53	0.1610	0.6072		0.3620	0.7833		0.9420	1.0028
Ribitol-5TMS	-0.17 ± 0.65	0.59 ± 0.49	-0.84 ± 1.27	0.42 ± 0.59	0.0005	0.0628	*	0.0069	0.0825	*	0.0259	0.8546
Ribonolactone-3TMS	-0.10 ± 0.60	0.08 ± 0.67	-0.61 ± 1.17	0.63 ± 0.96	0.1250	0.6111		0.8940	0.9834		0.4290	0.9134
Ribose-5-phosphate-meto-5TMS(1)	-0.62 ± 0.63	-0.70 ± 0.87	0.48 ± 0.72	0.84 ± 0.65	0.4120	0.8772		0.5200	0.8270		0.6400	0.9600
Ribulose-meto-4TMS	-0.83 ± 0.68	0.29 ± 0.35	-0.18 ± 0.60	0.72 ± 1.28	0.1735	0.6190		0.0174	0.1531		0.2436	1.0049
Serine-3TMS	0.37 ± 1.40	-0.64 ± 0.30	0.82 ± 0.78	-0.55 ± 0.10	0.5730	0.8498		0.0591	0.2786		0.8713	1.0001
Sorbitol-6TMS	0.92 ± 1.24	0.23 ± 0.75	-0.53 ± 0.27	-0.63 ± 0.60	0.7660	0.9722		0.9530	1.0064		0.3840	0.9564
Sorbose-meto-5TMS(1)	0.93 ± 1.29	0.01 ± 0.89	-0.40 ± 0.46	-0.55 ± 0.40	0.0418	0.6131		0.6483	0.9104		0.5277	0.9675
Spermidine-5TMS	0.35 ± 0.74	-0.41 ± 0.32	0.09 ± 1.68	-0.03 ± 0.35	0.1115	0.5661		0.7266	0.9222		0.0885	1.1682
Stearic-acid-TMS	0.38 ± 1.25	0.20 ± 0.56	-0.83 ± 0.62	0.26 ± 0.96	0.0524	0.4941		0.3168	0.7336		0.3527	0.9501
Succinic-acid-2TMS	-0.02 ± 1.25	0.12 ± 1.00	-0.37 ± 0.94	0.27 ± 0.67	0.1370	0.6236		0.3910	0.7480		0.5180	0.9630
Tagatose-meto-5TMS(2)	0.94 ± 1.29	0.03 ± 0.90	-0.41 ± 0.39	-0.56 ± 0.38	0.0571	0.4711		0.6555	0.9108		0.5730	0.9338
Threitol-4TMS	-0.70 ± 0.17	1.11 ± 0.74	-1.01 ± 0.14	0.59 ± 0.34	0.5775	0.8377		0.0009	0.0201	*	0.6629	0.9211
Threonic-acid-4TMS	-0.14 ± 1.04	-0.87 ± 0.66	0.28 ± 0.70	0.73 ± 0.78	0.6040	0.8573		0.3680	0.7590		0.1600	1.0560
Threonine-3TMS	0.39 ± 1.41	-0.66 ± 0.31	0.83 ± 0.72	-0.56 ± 0.08	0.5661	0.8589		0.0366	0.2196		0.8275	1.0305
Trehalose-8TMS	0.96 ± 1.12	0.42 ± 0.82	-0.66 ± 0.24	-0.71 ± 0.17	0.2110	0.6477		0.1050	0.3647		0.4080	0.9286
Tryptophan-3TMS	0.45 ± 1.21	-0.65 ± 0.79	0.37 ± 0.92	-0.17 ± 0.60	0.6510	0.8859		0.3770	0.7427		0.5870	0.9335
Tyrosine-3TMS	0.47 ± 1.26	-0.87 ± 0.60	0.77 ± 0.56	-0.37 ± 0.38	0.8766	0.9643		0.0349	0.2425		0.5445	0.9215
Uracil-2TMS	0.43 ± 1.51	-0.05 ± 0.71	-0.11 ± 0.88	-0.27 ± 0.65	0.0305	0.8052		0.1856	0.5568		0.5362	0.9565
Urea-2TMS	0.83 ± 1.04	-0.75 ± 0.17	0.50 ± 0.96	-0.58 ± 0.40	0.2076	0.6684		0.0103	0.1046		0.2017	1.1094
Ureidopropionic-acid-2TMS	-0.01 ± 1.05	0.27 ± 1.36	-0.58 ± 0.52	0.31 ± 0.54	0.5140	0.8077		0.7820	0.9558		0.8700	1.0074
Ureidosuccinic-acid-3TMS	0.50 ± 1.54	0.13 ± 0.75	-0.27 ± 0.42	-0.36 ± 0.84	0.6040	0.8482		0.2790	0.7082		0.1020	1.2240
Uric-acid-4TMS	1.23 ± 0.63	0.40 ± 0.55	-0.97 ± 0.31	-0.65 ± 0.23	0.0203	0.6699		0.7217	0.9340		0.3473	0.9551
Uridine-4TMS	0.08 ± 1.27	-0.44 ± 1.05	0.33 ± 0.87	0.03 ± 0.57	0.4460	0.8410		0.7340	0.9140		0.2490	0.9667
Valine-2TMS	0.54 ± 1.27	-0.77 ± 0.36	0.89 ± 0.49	-0.67 ± 0.10	0.7710	0.9693		0.0060	0.0880	*	0.9760	0.9910
Xanthine-3TMS	0.42 ± 1.30	0.33 ± 0.93	-0.79 ± 0.32	0.04 ± 0.76	0.7100	0.9372		0.9800	0.9951		0.9430	0.9958
Xylulose-meto-4TMS	-0.76 ± 0.69	0.00 ± 0.74	-0.35 ± 0.46	1.10 ± 0.86	0.0924	0.5544		0.1112	0.3580		0.2070	1.0930

Supplementary Table 3. Ingredient analysis of mixed feed and fermented feed[1].

Ingredient	Mixed feed	Fermented feed
Crude Protein (%)	17.50	20.60
Binding Protein (CP%)	3.50	3.40
Neutral-detergent Insoluble Protein (CP%)	13.20	20.90
Neutral-detergent Fiber (%)	13.00	16.20
Acid-detergent fiber (%)	5.90	6.40
Acid-detergent Lignin (%)	1.60	2.10
Starch (%)	45.00	37.50
Nonfibrous Carbohydrate (%)	51.90	46.80
Crude Fat (%)	5.90	6.90
Crude Ash (%)	14.00	13.80
Calcium (%)	4.06	4.69
Phosphate (%)	0.54	1.22
Magnesium (%)	0.19	0.43
Potassium (%)	0.73	1.10
TDN (%)	76.50	76.90
NE l (Mcal/kg)	1.76	1.81
NE m (Mcal/kg)	1.88	1.92
NE g (Mcal/kg)	1.24	1.28
Cell content (%)	76.70	72.30
Total Fiber (%)	9.40	13.90
Arginine (%)	0.66	0.85
Glycine (%)	0.84	1.39
Histidine (%)	0.39	0.33
Isoleucine (%)	0.65	0.85
Leucine (%)	1.62	1.49
Lysine (%)	0.96	0.87
Methionine (%)	0.35	0.34

Supplementary Table 3. (Continued)

Phenylalanine (%)	0.83	0.95
Tyrosine (%)	0.07	0.17
Valine (%)	0.81	1.11
Serine (%)	0.85	0.93
Alanine (%)	1.02	1.56
Aspartic Acid (%)	1.30	1.36
Glutamic Acid (%)	2.80	2.99
Proline (%)	1.22	1.35
Threonine (%)	0.71	0.76
Water (%)	11.90	36.20
Vitamin A (β-carotene) (IU/kg)	171.70	2321.00

[1]% means percentage in dry matter.
